# Sponge Gold

**Published:** 1852-01

**Authors:** 


*Sponge Gold.*—In the October number of the Journal, for 1850, we took occasion to call the attention of our readers to a new preparation of gold for filling teeth, and in the first number of the second volume of our New Series, we copied from the *London Medical Gazette,* an article, written by Mr. Tomes, Surgeon Dentist to the Middlesex Hospital, in which was described two forms of gold, made by Mr. Makins, which he had employed with the most satisfactory results. Wishing to try these new preparations, we sent to Mr. Ash, of London, for a small quantity, some of which, he at once forwarded to us by express. But as it does not correspond in appearance, with either of the preparations described by Mr. Tomes, as having been made by Mr. Makins, we fear he has sent, through mistake, another description of gold, one also referred to by Mr. Tomes. It consists of small irregular masses, of a porous texture, and of a light brown color, which are readily broken down into powder by pressure. These, however, and even the powder, are susceptible of being compressed into a solid mass. The experiments which we have made in filling teeth with it, have convinced us that a good filling can be made with it. Its use, however, for this purpose, is attended with considerable difficulty, in filling a cavity in the approximal surface of a tooth, and, indeed, in filling a cavity in any part of a tooth in the upper jaw, in consequence of its tendency to crumble and escape into the mouth, before sufficient pressure can be applied to weld the particles together. This difficulty may be measurably overcome by wrapping gold foil around it, in the manner as suggested by Mr. Tomes. In this way, it may be very conveniently employed for filling large cavities in the grinding surfaces of the molar teeth, in either jaw, but for small cavities and cavities in the sides of teeth, it cannot be used as satisfactorily as gold foil, inasmuch as the envelope is liable to be perforated and broken before the contents are forced into the cavity, and the particles welded together.

This preparation of gold, however, may, in locations where it can be advantageously employed, be made to adapt itself to all the inequalities of the cavity, and when properly condensed, if the tooth be broken, it will come out in a solid mass, requiring considerable force to break it. The angles will be found to be sharp and more resisting than a filling of foil. When broken, the fracture presents a frosted appearance, as does also the sides which were in contact with the walls of the cavity, while the surface is the color of gold, and is susceptible of a very fine and beautiful finish.

So far as we have tried this preparation, we think it preferable to the one made in New York, but, without great improvement, it can never be made to take the place wholly of gold foil, though there may be some cases, perhaps, in which it can be used more advantageously. As soon as we can have some prepared, we intend to try the precipitated leaf and sponge, recommended by Mr. Tomes, and when we shall have done so, we will make our readers acquainted with the result.

